# Supplementary material for: Transcriptomic Insights into Mechanisms of Early Seed Maturation in the Garden Pea (Pisum sativum L.)
Source: Cells. 2020 Mar 23;9(3):779. doi: 10.3390/cells9030779 (PMC7140803; doi:10.3390/cells9030779)

Zhewan−1(10)

Global Summary

%DE = 0.86

genes with fdr < 0.2 = 532 (138 + / 394 −)

genes with fdr < 0.1 = 499 (121 + / 378 −)

genes with fdr < 0.05 = 473 (108 + / 365 −)

genes with fdr < 0.01 = 419 (81 + / 338 −)

genes in genesets = 0

<FC> = 0

<t−score> = −478.91

<p−value> = 0

<fdr> = 0.14

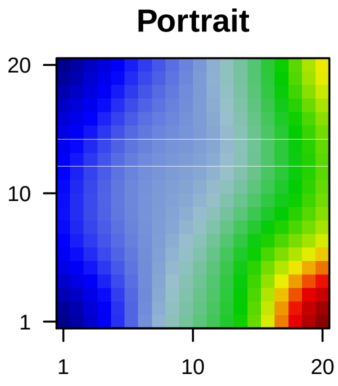

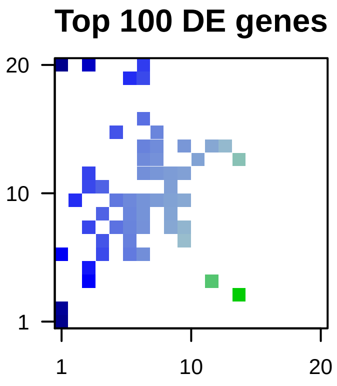

Global Genelist

Rank	ID	log(FC)	p−value	fdr	Metagene	Description
1	TRINITY_DN8	−0.71	3e−15	3e−11	1 x 1	
2	TRINITY_DN2	−0.19	4e−13	3e−11	1 x 2	
3	TRINITY_DN1	−0.07	8e−13	3e−11	3 x 4	
4	TRINITY_DN6	−0.05	1e−12	4e−11	6 x 6	
5	TRINITY_DN7	−0.08	1e−12	8e−11	1 x 6	
6	comp4770_c0	−0.02	3e−12	8e−11	7 x 13	
7	TRINITY_DN1	−0.02	3e−12	2e−10	10 x 7	
8	TRINITY_DN1	−0.04	6e−12	3e−10	3 x 8	
9	TRINITY_DN9	−0.03	1e−11	3e−10	12 x 14	
10	TRINITY_DN1	−0.07	1e−11	3e−10	6 x 19	
11	TRINITY_DN7	−0.03	1e−11	3e−10	3 x 11	
12	TRINITY_DN1	−0.02	2e−11	3e−10	7 x 6	
13	TRINITY_DN6	−0.03	3e−11	3e−10	4 x 7	
14	TRINITY_DN1	−0.02	3e−11	3e−10	4 x 9	
15	TRINITY_DN4	−0.06	3e−11	3e−10	14 x 13	
16	TRINITY_DN7	−0.02	3e−11	3e−10	4 x 11	
17	TRINITY_DN4	−0.04	3e−11	7e−10	4 x 6	
18	TRINITY_DN1	−0.03	5e−11	7e−10	5 x 15	
19	TRINITY_DN2	0	5e−11	8e−10	9 x 11	
20	TRINITY_DN1	−0.03	6e−11	8e−10	4 x 6	

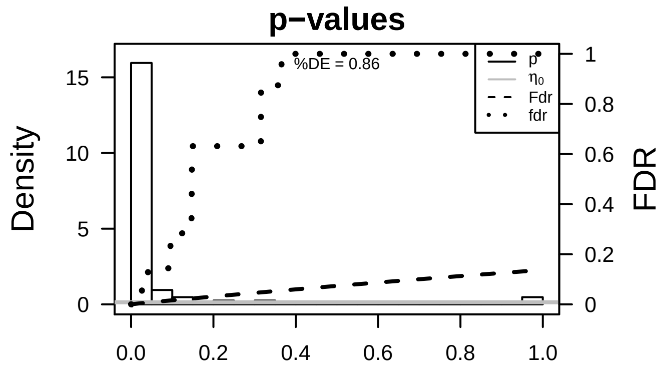

Supplement: Supplementary file 1 [file cells-09-00779-s001.zip › Supplementary materials/FileS3.zip!/FileS2/Hormones - Results/Summary Sheets - Groups/Reports/Zhewan.1.10..pdf]
